# Adherence to Oral Nutritional Supplements: A Review of Trends in Intervention Characteristics and Terminology Use Since the Year 2000

**DOI:** 10.1002/fsn3.4722

**Published:** 2025-01-09

**Authors:** Malin Skinnars Josefsson, Sandra Einarsson, Linn Seppälä, Liz Payne, Lisa Söderström, Evelina Liljeberg

**Affiliations:** ^1^ Department of Food Studies, Nutrition and Dietetics Uppsala University Uppsala Sweden; ^2^ Department of Food, Nutrition and Culinary Science Umeå University Umeå Sweden; ^3^ Pediatric Clinic at Umeå University Hospital Region Västerbotten Sweden; ^4^ School of Psychology University of Southampton Southampton UK; ^5^ Centre for Clinical Research Västerås Uppsala University Västerås Sweden; ^6^ Geriatrics, Rehabilitation Medicine and Pain Centre Uppsala University Hospital Uppsala Sweden; ^7^ Department of Women's and Children's Health Uppsala University Uppsala Sweden

**Keywords:** adherence, adherence assessment, compliance, nutrition intervention, oral nutritional supplement

## Abstract

Research on disease‐related malnutrition and adherence to oral nutritional supplements (ONS) has increased in recent years. To guide future studies, it is important to identify trends in terminology use and intervention characteristics. This review aimed to map characteristics of research investigating adherence to ONS in patients with disease‐related malnutrition and explore changes over time. This review is a secondary analysis of quantitative studies from a systematic mixed‐studies review. Online databases, including PubMed, Cinahl, Cochrane Central Register of Controlled Trials, and APA PsycInfo, were searched to identify studies published from 2000 to March 2022. A quantitative content analysis of extracted data was performed, and the Mixed Methods Appraisal Tool (MMAT) was used to assess methodological risk of bias. This review includes 137 articles, over half of which are randomized controlled trials (52%). The term “oral nutritional supplements” was used in 40% of the studies. Adherence to ONS was mainly described by the term “compliance” (69%). It was most common to offer ready‐made milk‐based ONS (56%) and ONS as a sole intervention (51%). The prescribed dose of ONS was fixed in 64% of studies and individualized in 22% of studies. There was variation in the methods used to assess adherence to ONS, and adherence was not reported in nearly a fifth of studies. There was an increase in methodological quality over time (*p* = 0.024). To ensure better understanding and increase the rigor and reproducibility of ONS intervention research, it is crucial to standardize the terminology used and to describe the interventions clearly.

## Graphical abstract

This review, encompassing 137 articles, mapped the characteristics of research on adherence to oral nutritional supplements (ONS) in patients with disease‐related malnutrition and explored changes over time. Key findings reveal a diverse range of terms used to describe ONS and adherence to this nutrition therapy. The ONS interventions frequently deviated from appropriate prescribing descriptions in nutrition guidelines, and there was a modest improvement in methodological quality over time.

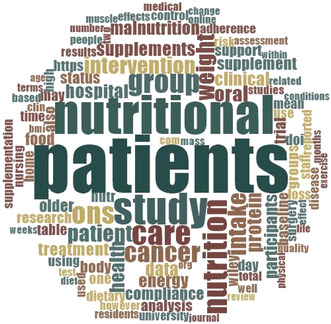

## 1 Introduction

Disease‐related malnutrition, also known as undernutrition, is associated with adverse health outcomes that negatively affect individual patients as well as the healthcare system (Marshall, Bauer, and Isenring 2014; Saunders and Smith 2010; Scholes 2022). Nutrition therapy is crucial in the treatment of malnutrition, and oral nutritional supplements (ONS) have become an essential treatment component across various medical disciplines (Arends et al. 2017; Bischoff et al. 2023; Collins, Yang, et al. 2019; Ikizler et al. 2020; Volkert et al. 2019; Weimann et al. 2021).

Many studies have concluded that interventions using ONS have a positive effect on the health of study participants (Cawood, Elia, and Stratton 2012; Deutz et al. 2021; Lauque et al. 2004; Tangvik et al. 2021; Weiner et al. 2014), although some reviews have reported uncertainty about the beneficial effects (Baldwin et al. 2021; Mello et al. 2021). Low adherence to ONS has been reported in some populations (Grass et al. 2015; Skladany et al. 2021; Wan et al. 2021) and might reduce the possibility of reaching the goals of nutrition therapy and lower the effect size of ONS in intervention studies (De Van Der Schueren et al. 2018). Also, a third of randomized controlled trials (RCTs) studying ONS do not report information on ONS adherence (Liljeberg et al. 2018), which may complicate the interpretation of study results. Hence, reviews have reported not only varying effects of ONS but also low‐quality evidence (Baldwin et al. 2021). Another limitation in previous studies is the incomplete descriptions of ONS interventions. For instance, Liljeberg et al. (2018) found that out of 76 RCTs, just 3% provided complete details of the ONS intervention. Comprehensive descriptions are necessary to accurately understand and interpret the effects of nutrition interventions.

Research into disease‐related malnutrition and adherence to nutrition therapy with ONS has increased during the past 20 years. As with all biomedical specialties, the large research output creates a greater need for reviewing and summarizing previous research to support best practice and to identify and answer important new questions (Mulrow 1994). The terminology used and reporting completeness of empirical studies are of great importance to enable their inclusion in reviews (i.e., by capturing all relevant publications) and to build on previous research (i.e., by understanding what has been done) (Hoffmann et al. 2014; Mulrow 1994).

In previous studies of adherence to medical treatment, the terms compliance and adherence are used interchangeably. For example, different terms have been used to describe deviation from prescribed medication regimens, and the term used has changed over time (1961–2009) (Vrijens et al. 2012). The authors argue that uniformity in terminology is needed, and they present a new taxonomy for describing and defining adherence to medications. In addition, the World Health Organization (WHO) emphasizes the need to differentiate between the terms compliance and adherence (Sabaté 2003).

Compliance refers to the extent to which a patient's behavior aligns with clinical prescriptions, such as taking medications, following diets, or making other lifestyle changes (Haynes and Sackett 1976). It reflects the patient's obligation to comply with the healthcare provider's instruction. Adherence, on the other hand, refers to the patient's willingness to accept the clinical prescription and actively involves the patient in the prescription process. Adherence is defined by WHO in the following way: “The extent to which a person's behavior – taking medication, following a diet, and/or executing lifestyle changes, corresponds with agreed recommendations from a health care provider” (Sabaté 2003).

Although the terminology used in studies related to ONS varies, ONS treatment has been clearly defined. The 2017 European Society of Clinical Nutrition and Metabolism (ESPEN) guidelines aimed to establish a consensus on nutrition‐related concepts and processes (Cederholm et al. 2017). These guidelines defined ONS as a category of products called “food (for) special medical purposes” (FSMP) and further as products: “…developed to provide energy and nutrient dense solutions that are provided as ready to drink liquids, cremes or powder supplements that can be prepared as drinks or added to drinks and foods.” The terms used to describe ONS and ONS intervention characteristics in studies have not been summarized before, and such a summary could make trends visible and guide future research within this field.

Determining whether patients have adhered to the prescribed amount of ONS presents a challenge when interpreting research on ONS, as inadequate reporting in intervention studies makes it unclear how adherence to ONS interventions impacts results. Therefore, to guide future research, it is crucial to summarize and identify trends in terminology use and the characteristics of studies related to ONS adherence.

The aim of this review was to map characteristics of research investigating adherence to ONS in patients with disease‐related malnutrition and explore changes over time, more specifically to (i) map ONS intervention characteristics (e.g., type of ONS used, prescribed amount of ONS, and assessment method of adherence to ONS) and (ii) map the terminology used for ONS and adherence.

## 2 Methods and Materials

### 2.1 Study Design

The present review is a secondary analysis based on studies from a systematic mixed‐studies review (the original study) that aimed to describe barriers and facilitators to adherence to ONS among patients with disease‐related malnutrition or at nutritional risk (Liljeberg et al. 2024). The original study included quantitative, qualitative, and mixed‐methods studies. For the present review, only quantitative articles and mixed‐method studies from the original study were considered suitable for inclusion. The study protocol was published in Prospero in November 2021 (registration number CRD42021286200) (Liljeberg et al. 2021). The term adherence is used in the present review since it is the preferred term according to WHO. However, when referring to other studies, the term used by the authors is applied.

### 2.2 Search Strategy

For the original study, the following Population Intervention Comparison Outcome (PICO) criteria were used: (P) Patients, ≥ 18 years, with malnutrition or at nutritional risk due to disease or medical condition; (I) Nutrition therapy, including multi‐nutrient ONS with ≥ 2 macronutrients and micronutrients, liquid or other texture, ready‐made or home‐made ONS, administered orally and not by tube feeding; (C) Any comparator or no comparator; and (O) Factors affecting adherence to and/or usage of ONS, barriers and facilitators to ONS adherence/usage.

The search for articles was conducted with help from librarians at Uppsala University, Sweden, and included articles published from January 2000 to March 2022 from the following databases: PubMed, Cinahl, Cochrane Central Register of Controlled Trials, and APA PsycInfo. The initial search identified 29,360 articles, and after removing duplicates, 21,835 articles were screened. The first screening was conducted by two authors (EL and SE) reading titles and abstracts, using the Rayyan tool (Ouzzani et al. 2016). The full text of 507 of the screened articles was read by the same two researchers, and 171 articles were included in the mixed‐studies review (original study). After removing articles using qualitative study design, 137 articles were included in the present review (PRISMA diagram in Appendix 1).

### 2.3 Risk of Bias Assessment

A risk of bias assessment (Furuya‐Kanamori et al. 2021) of the included articles was carried out by two other researchers (LSö and MSJ) using the Mixed Methods Appraisal Tool (MMAT version 2018) (Hong et al. 2019). The MMAT consists of a checklist and a user guide and can be used to assess the methodological quality of quantitative, qualitative, and mixed‐methods studies. MMAT includes five core criteria for quantitative study designs. There are three sets of criteria (qualitative, quantitative, and mixed) for mixed‐method studies, and the lowest score from these criteria constitutes the overall score for this study design. Each criterion is marked with yes, no, or can't tell. Every yes is then given 20 percentage scores. Hence a study can vary between 0% and 100%, and the higher the score, the lower the risk of bias. In the original and the present review, articles were included in the analysis regardless of the MMAT result, since the aim of the risk of bias assessment was to evaluate and reveal the quality of all articles.

### 2.4 Data Extraction and Analysis

A quantitative content analysis of extracted data was performed (Clark et al. 2021), starting by creating a coding manual and an Excel file into which relevant data from the included articles were extracted. The coding manual contained 11 items of information (10 categorical and one quantitative variable). The items represented general aspects of the studies, ONS characteristics, and information about adherence assessment and the terminology used. For each categorical item in the variable list, the response options were coded (assigned with a number).

A pilot study was conducted using the first version of the coding manual. In the pilot study, the coding manual and its codes were used to assess 13 (10%) of the included articles. The purpose of the pilot study was to assess whether new variables or codes should be added or if existing variables or codes should be retained, removed, or modified. The chosen articles had different study designs so that the final coding manual would fit as many study designs as possible. Three of the authors (LSe, EL, and SE) conducted the pilot study, and discussions were held to reach consensus when there was disagreement in the variables and/or codes. The variables included in the final coding manual are presented in Table 1. After the final version of the coding manual was finished, one author (LSe) extracted data from the remaining articles.

**TABLE 1:** The included variables in the final coding manual.

Study characteristics	Oral nutritional supplements (ONS)	Adherence
Year of publication	Used term for ONS	Used term for adherence
Study design	ONS intervention^a^	Assessment method of ONS intake or adherence
Country of origin	Prescription dose of ONS	How adherence rate was reported
Patient group/diagnosis	Style of ONS^b^	

^a^ For example, if the intervention included only ONS or ONS in combination with another treatment component.

^b^ For example, if the ONS in the study was milk‐based or juicy.

Data were converted and analyzed using the statistical program IBM SPSS Statistics version 28 (IBM Corp.). The results are presented using descriptive statistics, and Chi‐square and Spearman's rho were used for statistical analyses. Some of the variables were grouped before conducting the analyses, for example, the years of publication were divided into groups. A *p*‐value < 0.05 was considered statistically significant.

## 3 Results

### 3.1 Characteristics of Included Articles

**FIGURE 1:**
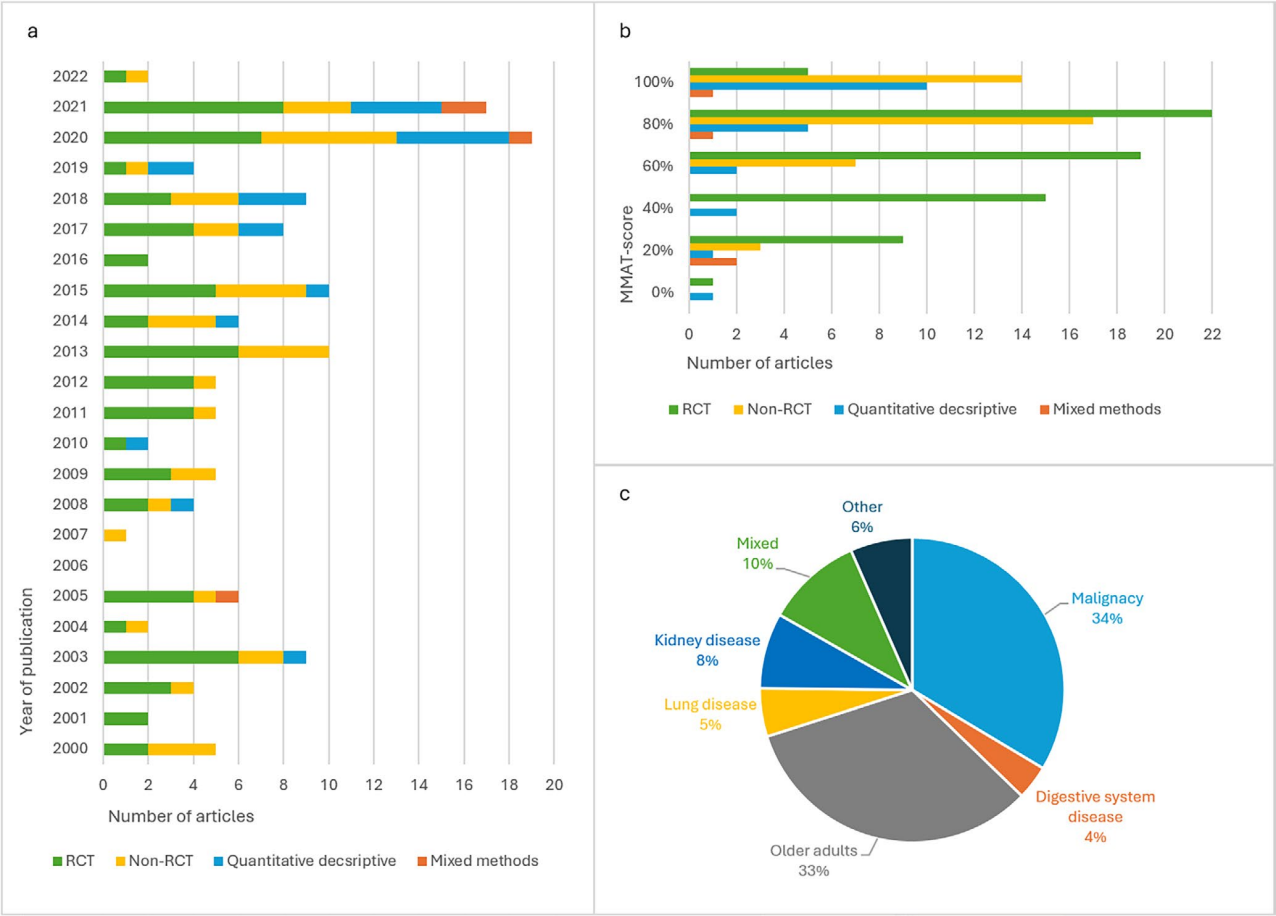
Characteristics of included articles, *n* = 137. (a) The number of included articles published each year from January 2000 to March 2022, divided by study design. (b) Study design and mixed‐method appraisal tool (MMAT)‐score risk of bias. High percentage indicates high quality. (c) Distribution (%) of patient population in the included articles.

In total, 137 articles were analyzed (Aldhahir et al. 2021; Allen, Methven, and Gosney 2014; Baldwin et al. 2011; Bauer et al. 2005; Bauer, Isenring, and Waterhouse 2013; Beck, Ovesen, and Schroll 2002; Beck, Damkjær, and Tetens 2009; Beck et al. 2013; Bell et al. 2014; Brindisi et al. 2020; Berk et al. 2008; Boisselier et al. 2020; Bojesen et al. 2022; Bonnefoy et al. 2003; Breedveld‐Peters et al. 2012; Brown et al. 2020; Bruce et al. 2003; Caglar et al. 2002; Calder et al. 2018; Calegari et al. 2011; Cameron et al. 2011; Campbell et al. 2013; Cereda et al. 2015; Chapman et al. 2011; Citty et al. 2017, 2021; Collins, Tucker, et al. 2019; Cornejo‐Pareja et al. 2021; Cruz‐jentoft et al. 2008; de Luis et al. 2008; de Luis et al. 2015; de Oliveira Faria, Howell, et al. 2021; Dedeyne et al. 2018; den Uijl et al. 2015; Doll‐Shankaruk, Yau, and Oelke 2008; Enriquez‐Fernandez et al. 2022; Faccio et al. 2021; Fearon et al. 2003; Fiatarone Singh et al. 2000; Førli et al. 2001; Gazzotti et al. 2003; Ginzburg et al. 2018; Gosney 2003; Grass et al. 2015; Grönstedt et al. 2020; Hanai et al. 2018; Hashizume et al. 2019; Hertlein et al. 2018; Ho and Norshariza 2017; Hogan, Solomon, and Carey 2019; Hopanci Bicakli et al. 2017; Huang et al. 2020; Hübner et al. 2012; Hulsbæk et al. 2023; Hung et al. 2005; Ida et al. 2017; Imamura et al. 2021; Ishiki et al. 2015; Jackson et al. 2015; Jeloka et al. 2013; Jobse et al. 2015; Jukkola and MacLennan 2005; Karlsson et al. 2021; Keithley et al. 2002; Kobayashi et al. 2017; Kong et al. 2018; Kraft et al. 2012; Lad, Gott, and Gariballa 2005; Lambert et al. 2014; Lammel Ricardi et al. 2013; Lauque, Arnaud‐Battier, and Mansourian 2000; Laviano et al. 2020; Lawson et al. 2021; Lawson et al. 2000; Lidoriki et al. 2020; Liljeberg et al. 2019; Lombard et al. 2014; Malafarina et al. 2021; Mantovani et al. 2004; Martin et al. 2019; Daud et al. 2012; Mayr et al. 2000, 2016; McCormick et al. 2007; McDermott et al. 2003; McMurdo et al. 2009; Meade 2007; Miller et al. 2005; Myers 2010; Nasrah et al. 2020; Neoh et al. 2020; Olde Rikkert et al. 2015; Palma‐Milla et al. 2018; Pastore, Orlandi, and Gonzalez 2014; Patursson et al. 2021; Percival et al. 2013; Pison et al. 2011; Planas et al. 2005; Previtali et al. 2020; Qin et al. 2022; Roberts et al. 2003; Rondanelli et al. 2020; Salamon and Lambert 2018; Sandmæl et al. 2017; Schmidt et al. 2020; Scott et al. 2009; Seemer et al. 2020; Seguy et al. 2020; Sharma et al. 2002; Shirakawa et al. 2012; Skladany et al. 2021; Smith et al. 2020; Solheim et al. 2017; Stange et al. 2013; Steiner et al. 2003; Storck et al. 2020; Stow, Smith, and Rushton 2018; Taib et al. 2021; Tanaka et al. 2018, 2021; Trachootham et al. 2015; Uí Dhuibhir, Collura, and Walsh 2019; van den Berg, Lindeboom, and van der Zwet 2015; van der Meij et al. 2010; Verma et al. 2000; Verma, Holdsworth, and Giaffer 2001; Vermeeren et al. 2004; Wall et al. 2020; Wan et al. 2021; Weenen et al. 2014; Wengstrom, Wahren, and Grodzinsky 2009; Wong et al. 2021; Wu et al. 2013; Xie et al. 2021; Young, Banks, and Mudge 2018; Zak, Swine, and Grodzicki 2009; Zhang et al. 2022) (Appendix 2). The study designs were mainly RCTs, 71/137 (52%), and non‐RCTs, 41/137 (30%). There were 21/137 (15%) articles that used a quantitative descriptive design and 4/137 (3%) using mixed methods. One example of a mixed‐methods study design was a study that quantitatively collected information about patients' intake of and adherence to ONS and qualitatively collected information about attitudes and views on ONS (Brindisi et al. 2020). About one‐third of the articles, 45/137 (33%), were published between the years of 2000 and 2011, and about two‐thirds were published between 2012 and March 2022, 92/137 (67%) (Figure 1a).

The median MMAT score for the articles over the studied period was 80%, and 30/137 (22%) scored 100% (Figure 1b). Among the RCTs, 5/71 (0.7%) scored 100%. The equivalent share among other study designs was: non‐RCTs 14/41 (34%); descriptive studies 10/21 (48%); and 1/4 (25%) for mixed‐method studies. The median MMAT score for articles published between 2000 and 2011 was 60%, and for those published from 2012 to 2022, 80%. Hence, there was a positive increase of the MMAT scores over time (rho = 0.193, *p* = 0.024).

Of the included articles, 77/137 (57%) involved outpatients, 40/137 (29%) inpatients, and 19/137 (14%) a mixture of both (Appendix 2). Further, 45/137 (33%) studies involved older adults and 46/137 (34%) patients with malignancy (Figure 1c). The others presented studies on patients with kidney diseases 11/137 (8%), patients with digestive system diseases 5/137 (3%), patients with lung diseases 7/137 (5%), and patients with other diseases, for example, wounds or fractures, 9/137 (7%). Fourteen (10%) articles included patients with two or more of the aforementioned diagnoses. Of the articles on older adults (*n* = 45), 60% involved inpatient settings such as nursing homes and hospitals, while only 2% of the articles on patients with malignancies represented inpatient settings.

**FIGURE 2:**
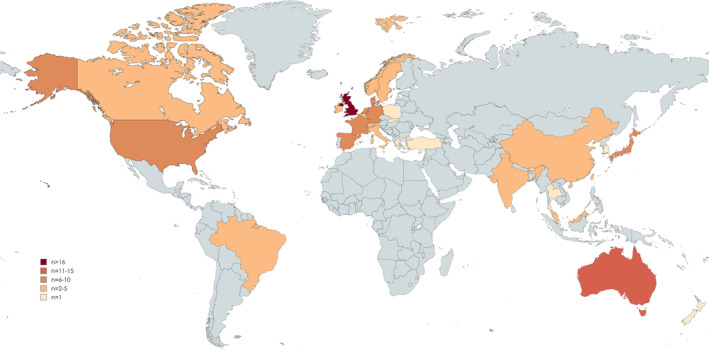
Distribution of included articles (*n* = 137) by country. Map created in mapchart.net.

Most of the studies were conducted in Europe (19 countries), 82/137 (59%) (Figure 2 and Appendix 2). Twenty‐three studies (17%) were conducted in Asia, and 15/137 (11%) studies in Oceania. North America contributed 12/137 (9%) studies, and South America 5/137 (4%) studies.

### 3.2 Oral Nutritional Supplement Intervention Characteristics

**FIGURE 3:**
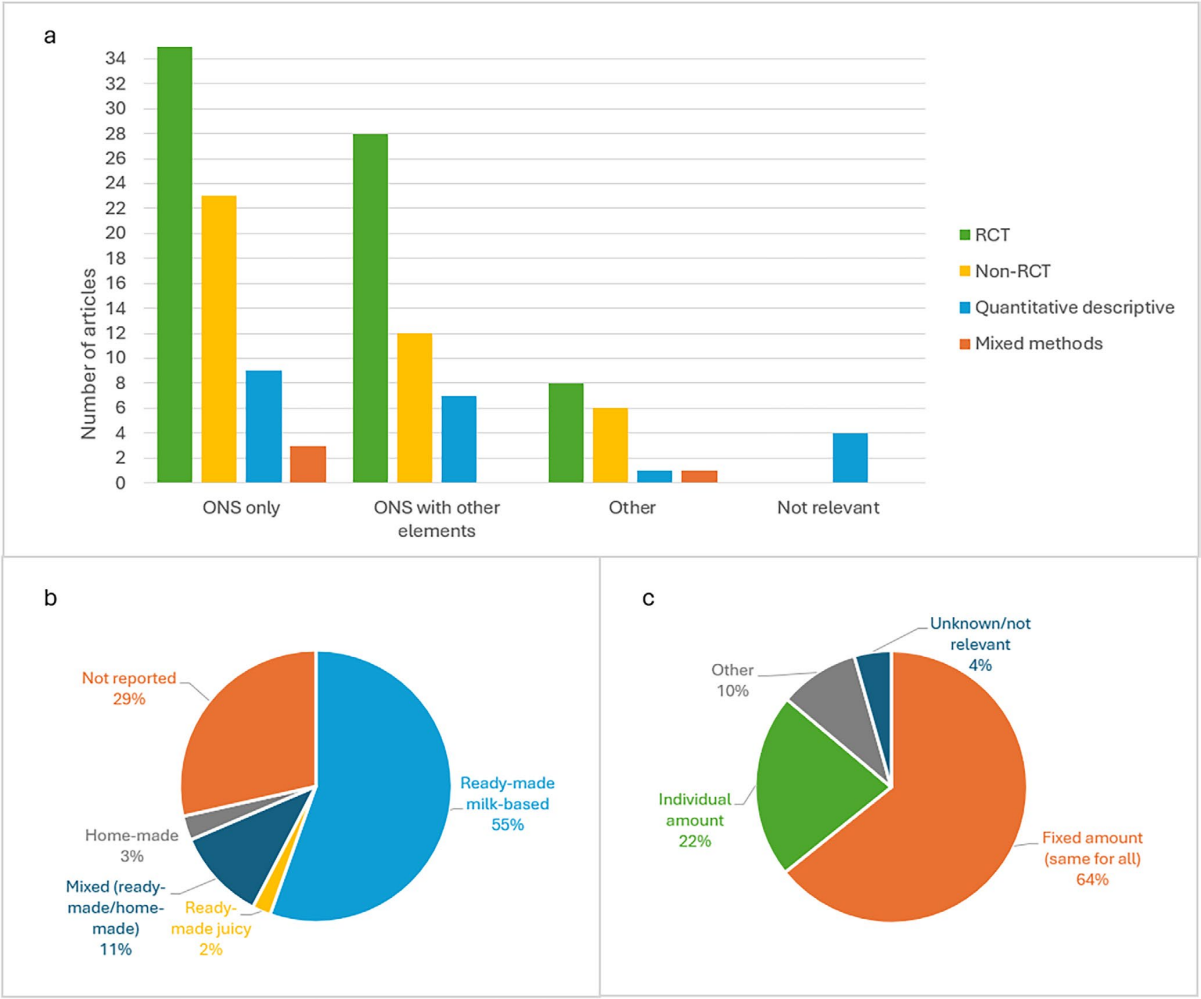
Oral nutritional supplement (ONS) intervention characteristics in included articles, *n* = 137. (a) Distribution of ONS intervention contents and study design. (b) Distribution (%) of intervention type. (c) Distribution (%) of intervention dose.

In the studies, across all study designs, it was most common to offer ONS as a sole intervention 70/137 (51%) (Figure 3a). In the RCTs, 35/71 (49%) used ONS as the sole intervention, while 28/71 (39%) used ONS in combination with other elements, e.g., nutrition counseling and/or physical activity or multiple different elements combined with ONS in two or more arms. An example of interventions classified as “other” included ONS in several combinations with, e.g., wound care, physical activity, texture modification, and nutritional counseling. One example of an article categorized as a multiple‐armed RCT combined different types of physical exercise with ONS or placebo into four different intervention groups (Zak, Swine, and Grodzicki 2009).

Further, the style of ONS used in the interventions was ready‐made milk‐based products 76/137 (56%), a mix of ready‐made and home‐made products 15/137 (11%), ready‐made juice products 3/137 (2%), and home‐made ONS 4/137 (3%), while the type of ONS was not reported in 39/137 (28%) of the articles (Figure 3b). Among the studies using ONS as a sole intervention, ready‐made milk‐based ONS was most common and used in 40/70 (57%) studies.

The prescribed doses of ONS were fixed (i.e., the same amount of ONS was given to all participants) in 88/137 (64%) of the studies (Figure 3c). In 30/137 (22%) of the articles, the patients received a prescribed dose of ONS related to their individual needs, e.g., adjusting the ONS amount to ensure the patient reached an intake of 30 kcal/kg/day (Huang et al. 2020). In 13/137 (10%) of the articles, the prescription dose was based on something else, e.g., it could vary according to the level of malnutrition or energy deficiency in subgroups. In the remaining articles 6/137 (4%), ONS prescription was not reported or reported as not relevant, i.e., the prescribed doses were not relevant or necessary for the aim of the study.

### 3.3 Terms Used for Oral Nutritional Supplement and Adherence

**FIGURE 4:**
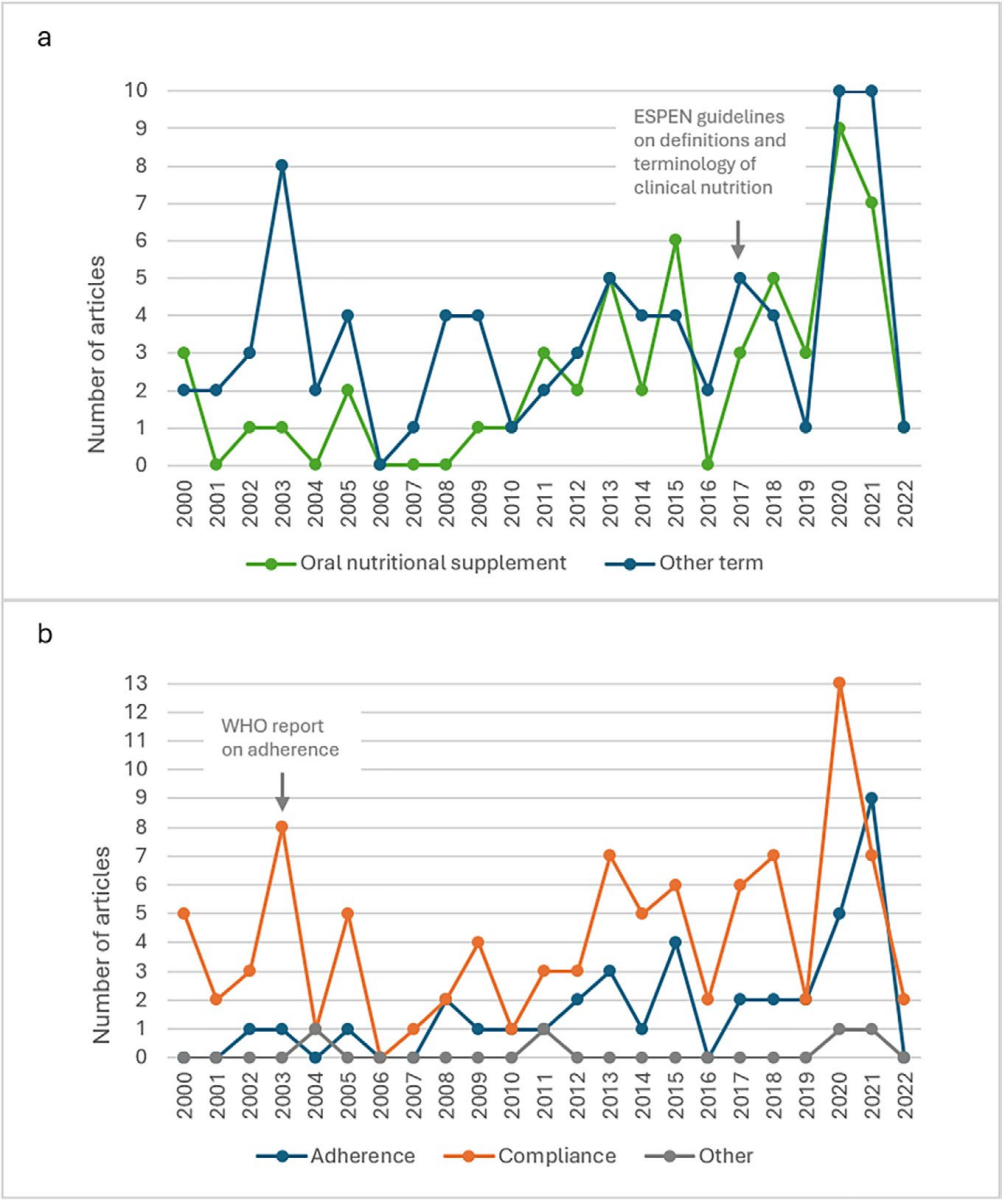
Terminology used in included articles published between January 2000 and February 2022, *n* = 137 (a). Terminology used for “oral nutritional supplement”. (b) Terminology used for adherence to oral nutritional supplements.

The term “oral nutritional supplement” (or equivalents, e.g., “oral nutrition supplements” and “oral nutritional supplementation”) was used to describe the ONS used in 55/137 (40%) articles. The remaining 82 articles used other terms, e.g., “immunonutrition,” “protein supplementation,” or “protein‐energy supplement” (Appendix 3). As described earlier, ESPEN published a guideline in 2017 suggesting using the term “oral nutritional supplement” (Cederholm et al. 2017). Among the included articles, the proportion using “oral nutritional supplement” (or equivalents) was 35% (27/78) between 2000 and 2016, while the corresponding number from 2017 onwards was 47% (28/59) (Figure 4a). The use of the term ONS between the two periods showed no statistically significant difference (*p* = 0.129).

Among the included articles, “compliance” was the most frequently used term, 95/137 (69%), followed by “adherence” 38/137 (28%). In four articles (3%), other terms were used. The use of “compliance” was particularly notable in RCTs where 75% (60/80) used this term compared to 23% (18/80) using “adherence.” Two of the RCTs used other terms. As stated in the background, WHO published a report in 2003 clearly stating the need to differentiate between the two terms compliance and adherence, and preferably to use adherence^25^. During the years 2000–2003, the proportion using “compliance” was 90% (18/20), and 10% (2/20) used “adherence.” From the year 2004 and onwards, the corresponding shares for “compliance” and “adherence” were 66% (77/117) and 34% (40/117), respectively (Figure 4b). When comparing the use of “compliance” versus “adherence” (*n* = 133, “others” removed), among the articles published during the first half (2000–2011) and those published during the second half (2012–2022) of the studied period, the term “adherence” was used to a greater extent than “compliance” during the second half (*p* = 0.040).

### 3.4 Assessment of Oral Nutritional Supplement Adherence

**FIGURE 5:**
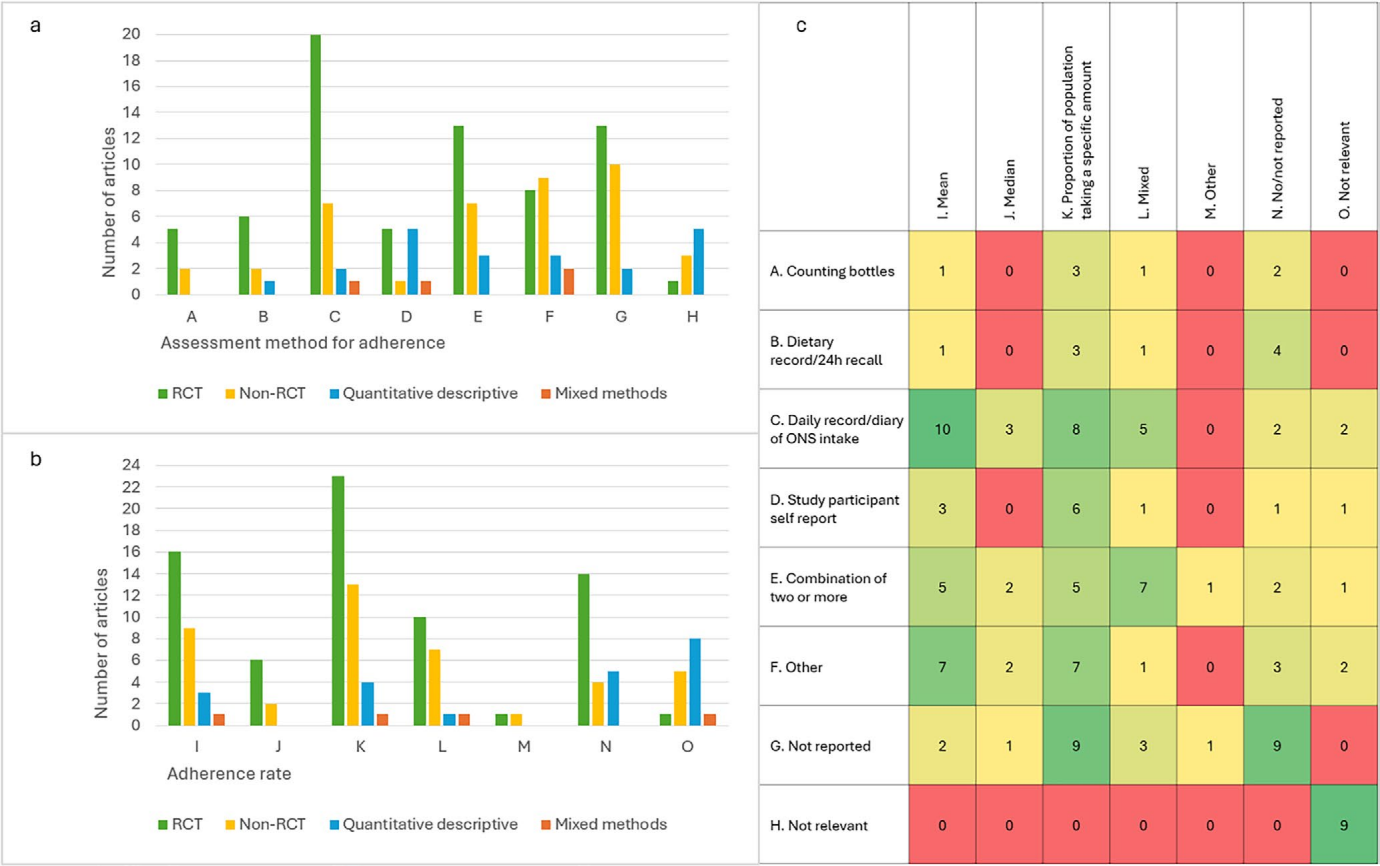
Assessment of oral nutritional supplement (ONS) adherence or intake in includes articles, *n* = 137. (a) Distribution of assessment method for adherence or intake of ONS by study design. (b) Distribution of adherence rate by study design. (c) Heat map ranging from red to green with increasing frequency of the distribution of assessment method of ONS intake and reporting of the adherence rate. Presented in frequencies.

A wide variety of methods of assessing ONS adherence (or intake) and ways of reporting the adherence rate were used in the articles (Figure 5a–c). Adherence or intake of ONS was mainly measured through a daily record or diary of ONS intake in 30/137 (22%) articles or a combination of two or more methods in 23/137 (17%), such as combining counting bottles with self‐report. The assessment method was not reported in 25/137 (18%) articles in total and not reported in 15/80 (19%) of the RCTs.

Adherence rate was mainly reported as a proportion of the population taking a specific amount, 41/137 (30%), or the mean intake of ONS, 29/137 (21%) (Figure 5b). Adherence rate was not reported in 23/137 (17%) of studies. When adherence was assessed through a daily record or diary of ONS intake, the adherence rate was reported as mean intake in 10/29 (33%) of studies (Figure 5c). In 16/137 (12%) articles, the adherence rate was reported while the assessment method was not reported. On the other hand, 14/137 (10%) articles described an assessment method but did not report the adherence rate.

## 4 Discussion

In the present review, the majority of the included articles were RCTs from high‐income countries (*n* = 71/137). In most studies, ONS was used as a sole intervention component, primarily in the form of ready‐made milk‐based ONS. The most common measurement method for adherence was to use diaries or daily records of ONS intake, and the adherence rate was most frequently reported as a proportion of the population taking a specific amount of ONS. The terminology on ONS and adherence to ONS differed between the studies, with more studies using the term “compliance” instead of “adherence,” especially in studies with experimental study designs such as RCTs. Though the methodological quality of studies significantly increased over time, information on important intervention aspects was still missing in many studies.

### 4.1 Oral Nutritional Supplements: Time for A Consistent Terminology Use

The majority of articles in the present review used terms other than “oral nutritional supplement.” The reason for diverse intervention terminology use might be time‐dependent trends, the existence of culturally specific terms/products, or the lack of a rationale for using a consistent terminology. For example, the quantity of literature review research has exploded during recent years, and the number of reporting guidelines is increasing (Equator network 2024; Smela et al. 2023). This has created a call for consistent terminology, which might not have received as much focus twenty years ago. In the Template for Intervention Description and Replication (TIDieR) checklist and guide, which is a reporting guideline applicable for all study designs, the guideline states that there should be a “precision in the name” that “enables easy identification of the type of intervention and facilitates linkage to other reports on the same intervention” (Hoffmann et al. 2014).

In many of the articles in this review, the term “nutritional supplement” or “dietary supplement” was used to describe oral nutritional supplements for patients with malnutrition. This demonstrates that those terms can encompass various products. For instance, Amirtaheri Afshar et al. used both the terms “nutritional supplement” and “dietary supplement” in their randomized trial in patients with knee osteoarthritis to describe a bioactive component‐rich oil from the plant *Nigella sativa* (Amirtaheri Afshar et al. 2023). The term “dietary supplement” has also been used to describe specific innovative supplements in capsule form containing antioxidants or high‐potential bioactive compounds (Nemzer, Fink, and Fink 2014; Praengam et al. 2024). To address this issue, the publication by ESPEN in 2017 can be consulted, as it provides standardized terminology for nutritional interventions and their definitions (Cederholm et al. 2017). Following these guidelines can help mitigate the problem of inconsistent terminology. Consistent use of terminology in both research and clinical practice offers several advantages, including enhanced comparability and credibility in the field of nutritional interventions. Therefore, we suggest as a solution to this problem that the term recommended by ESPEN—oral nutritional supplements (ONS)—should be the first choice for all scholars within this field. The term ONS can, of course, be combined with specific terms such as immunomodulation ONS or energy‐dense ONS, provided that the ONS term is always included together with the additional information. For example, Hubbard et al. found that compliance to “ONS with a higher energy density” was particularly beneficial (Hubbard et al. 2012). Therefore, it is important for authors to not only use the appropriate term for ONS but also to specify the content of the ONS to ensure a comprehensive understanding of the study context. From a wider perspective, authors should also be aware that the reporting of how nutritional care interventions for disease‐related malnutrition are individualized is inconsistent and lacks a consensus definition (Holdoway et al. 2022).

### 4.2 Compliance or Adherence? Situation‐Dependent Terminology Use

The term “compliance” was more commonly used than “adherence” in articles discussing ONS, and there was no apparent shift in terminology following the WHO report in 2003 (Sabaté 2003). Similarly, Vrijens et al. found that “compliance” and “adherence” were the most commonly used terms in studies on medication adherence published between 1961 and 2009 (Vrijens et al. 2012). However, it was suggested that the use of the term “compliance” would diminish over time, and they found an emphasis on the term “adherence” in later publications. In the present study, although “compliance” was still widely used, “adherence” was to some extent used more often than “compliance” during the second half (2012–2022) of the studied period.

The present review found that the term “compliance” was frequently used in RCTs/non‐RCTs, suggesting that the choice of terminology might be influenced by the study design. In clinical trials, the focus is commonly on efficacy, investigating the effect of an intervention under optimal conditions, including high adherence. This contrasts with real‐life healthcare systems, where patients are encouraged to play an active role in their treatment, and effectiveness is measured as an outcome (Costa et al. 2017).

In a qualitative study by Liljeberg et al. (2022), it was found that patients viewed the prescribed amount of ONS as a recommendation rather than an exact prescription, allowing them to adjust their intake accordingly. This suggests that patients exert some control over the prescription of ONS. In this context, the term “adherence” may be more appropriate than “compliance.” The definitions provided by WHO support this distinction, with “adherence” referring to patients playing an active role in the prescription process of ONS, while “compliance” implies a more passive role (Sabaté 2003). Hancock et al. (2012) demonstrated that patients prefer a guiding approach from dieticians rather than a commanding role, and that patients value the opportunity to actively participate in their treatment. These findings align with the principles of person‐centered healthcare, where patients are seen as experts in their own lives, and shared decision‐making is emphasized (Ewing et al. 2015). The WHO report on adherence underscores the importance of active patient participation in treatment (Sabaté 2003), which may suggest that use of the term “adherence” should be recommended in research in which ONS prescriptions are implemented in a person‐centered manner.

### 4.3 The Reporting of and Trends in Oral Nutritional Supplement Interventions and Adherence Assessment

The present review concluded that 28% of studies reported insufficient details about the style of ONS used. Similarly, 30% of RCTs published from 2002–2015 on ONS did not provide sufficient information regarding the specific style of ONS provided to the participants (Liljeberg et al. 2018). Among those that reported the ONS style, more than half of the articles in the present review used ready‐made milk‐based ONS. A review by Hubbard et al. in 2012, examined patients' compliance to ONS and influencing factors. Among the 46 studies included in their review, 96% focused on ready‐made, multi‐nutrient liquid ONS. It can be inferred from the results of the present and previous reviews that ready‐made ONS is the preferred ONS style to use in intervention studies. However, to be able to replicate a previous study in research or clinical practice and to interpret the results correctly, the reporting of the style of ONS needs to be improved.

In the present review, the majority of studies used a fixed amount of ONS as the sole intervention. This practice is not in line with nutrition guidelines and the fundamentals of person‐centred care (Cederholm et al. 2017). In nutrition guidelines, ONS are not suggested as a first‐line treatment, and they are typically recommended for use when nutrition counseling and food fortification are not sufficient to improve energy and nutrient intake (Arends et al. 2017; Ikizler et al. 2020; Volkert et al. 2019). Also, individualized nutrition goals should preferably guide the nutrition care plan. In the EFFORT trial, with over 2000 medical inpatient participants, individualized nutrition support, including tailored ONS prescriptions if needed, resulted in improved clinical outcomes and survival compared to the control group which received standard hospital food (Schuetz et al. 2019). Thereto, individualizing and adapting nutritional care to a patient's unique needs has been identified as an important aspect of person‐centred care within dietetic practice (Sladdin et al. 2017). This aligns with recommendations on appropriate ONS prescribing, where individual tailoring to patient preferences regarding type of ONS, time of consumption, and serving style are considered important for successful treatment (Stratton and Elia 2010).

In the present review, 18% of the included studies did not report adherence or intake of ONS, and 22% did not report how adherence was assessed. In the review conducted by Hubbard et al. (2012), it was found that 43% of the studies did not report the method used to measure compliance. Consequently, the reporting of measurement methods for ONS adherence seems to be frequently overlooked. To facilitate comparison of study results, it is of great importance that the measurement method for adherence is clearly stated. Furthermore, in their scoping review, de Oliveira Faria, Alvim Moravia, et al. (2021) discovered that researchers did not employ consistent measurement methods for ONS adherence, which is similar to the findings from the present review. Nutritional observational studies also commonly lack reports of important methodological details such as compliance rate and dietary assessment methods. Consequently, a specific reporting guideline for nutrition epidemiology studies exists, called Strengthening the Reporting of Observational Studies in Epidemiology—Nutritional Epidemiology (STROBE‐nut) (Lachat et al. 2016). To ensure clear and transparent research within this field, future studies should address the most appropriate way to measure adherence and report this sufficiently in order to establish a more unified understanding of adherence to ONS.

### 4.4 Strengths and Limitations

A strength of the current review is the inclusion of a large number of articles over a 20‐year period. The results can therefore serve as a guide for researchers when planning studies on ONS and adherence to ONS. In addition, varied study populations and interventions were included, which is important as it helps to fully understand the characteristics of research investigating adherence to ONS. However, it is important to consider that older adults and patients with malignancies were the two largest populations represented, which affects the generalizability of the results to other populations. There were also methodological differences between the studies included in the review, as it encompassed studies with adherence to ONS as the primary focus as well as studies that briefly mentioned ONS adherence somewhere in the text. This mixed set of data is a strength but should be interpreted through the lens of this broad inclusion strategy.

The codes and variables in the coding manual were modified not only before data extraction but also after the pilot analysis to ensure the best possible codes and variables for the dataset. As there were various ways to code the intervention contents, efforts were made to find the most logical and objective solutions suitable for the included articles. One person (LSe) coded the majority of the articles and no inter‐rater reliability test was made which are two drawbacks when it comes to quantitative content analysis (Clark et al. 2021). However, recurring consensus meetings were held (with EL and SE) on articles and variables that the primary coder could not easily code. However, the subjective nature of coding the variables is another important aspect to consider when interpreting the results.

## 5 Conclusion

The present review presents characteristics of research investigating adherence to ONS in patients with disease‐related malnutrition from a large number of articles over a 20‐year period. The main results were the widespread use of fixed amounts of ready‐made milk‐based ONS, the variation in the measurement methods used to assess adherence to ONS, and the inconsistent terminology employed, together with the slight increase in methodological quality over time. Many of the included articles used ONS as the sole intervention, while nutritional counseling together with individualized prescriptions of ONS is recommended in guidelines and in line with a person‐centered care approach. To ensure better understanding and increase the rigor and reproducibility of clinical nutrition research, it is of paramount importance to standardize the terminology and specify all details encompassing the ONS intervention. In accordance with this, the present review offers valuable insights for further research and can serve as a guide for researchers when planning studies on ONS and adherence to ONS.

## Author Contributions

**Malin Skinnars Josefsson:** conceptualization (equal), formal analysis (lead), investigation (lead), methodology (lead), validation (equal), visualization (equal), writing – original draft (lead), writing – review and editing (lead). **Sandra Einarsson:** conceptualization (lead), investigation (equal), methodology (equal), project administration (lead), validation (equal), writing – review and editing (lead). **Linn Seppälä:** conceptualization (supporting), formal analysis (equal), investigation (equal), methodology (equal), validation (equal), writing – review and editing (equal). **Liz Payne:** conceptualization (equal), methodology (equal), writing – review and editing (equal). **Lisa Söderström:** conceptualization (equal), investigation (equal), methodology (equal), writing – review and editing (equal). **Evelina Liljeberg:** conceptualization (lead), formal analysis (equal), investigation (equal), methodology (equal), project administration (lead), supervision (lead), validation (equal), visualization (equal), writing – review and editing (lead).

## Ethics Statement

The authors have nothing to report.

## Conflicts of Interest

The authors declare no conflicts of interest.

## Transparency Declaration

The lead author affirms that this manuscript is an honest, accurate, and transparent account of the study being reported. The reporting of this work is compliant with PRISMA guidelines. The lead author affirms that no important aspects of the study have been omitted and that any discrepancies from the study as planned have been explained.

## Data Availability

The data that support the findings of this study are available on request from the corresponding author.

## Appendix 2 Characteristics of Included Articles (*n* = 137)

**Table:**

Author	Year	Study design according to MMAT	Country	Diagnosis/Patient group	Setting^a^	MMAT‐score
Aldhahir et al.	2021	Randomized controlled trial	UK	Lung disease	Outpatient	80%
Allen et al.	2014	Randomized controlled trial	UK	Older adults	Inpatient	80%
Baldwin et al.	2011	Randomized controlled trial	UK	Malignancy	Outpatient	60%
Bauer et al.	2005	Randomized controlled trial	Multiple countries	Malignancy	Outpatient	20%
Bauer et al.	2013	Randomized controlled trial	Australia	Other (chronic wounds)	Outpatient	80%
Beck et al.	2002	Randomized controlled trial	Denmark	Older adults	Inpatient	20%
Beck et al.	2009	Randomized controlled trial	Denmark	Older adults	Inpatient	60%
Beck et al.	2013	Randomized controlled trial	Denmark	Older adults	Outpatient	60%
Bell et al.	2014	Non‐randomized study	Australia	Older adults	Inpatient	100%
Berk et al.	2008	Ra0ndomized controlled trial	USA	Malignancy	Outpatient	40%
Boisselier et al.	2020	Randomized controlled trial	France	Malignancy	Outpatient	100%
Bojesen et al.	2022	Non‐randomized study	Denmark	Malignancy	Outpatient	60%
Bonnefoy et al.	2003	Randomized controlled trial	France	Older adults	Inpatient	60%
Breedveld‐Peters et al.	2012	Randomized controlled trial	Netherlands	Older adults	Mixed	60%
Brindisi et al.	2020	Mixed methods study	France	Mixed diagnoses	Inpatient	20%
Brown et al.	2020	Quantitative descriptive study	UK	Older adults	Outpatient	60%
Bruce et al.	2003	Randomized controlled trial	Australia	Older adults	Outpatient	20%
Caglar et al.	2002	Non‐randomized study	USA	Kidney disease	Outpatient	80%
Calder et al.	2017	Randomized controlled trial	Sweden	Lung disease	Outpatient	80%
Calegari et al.	2011	Randomized controlled trial	Brazil	Kidney disease	Outpatient	40%
Cameron et al.	2011	Randomized controlled trial	Australia	Older adults	Outpatient	80%
Campbell et al.	2013	Non‐randomized study	Australia	Older adults	Inpatient	80%
Cereda et al.	2015	Randomized controlled trial	Italy	Other (pressure ulcers)	Mixed	80%
Chapman et al.	2011	Non‐randomized study	Australia	Other (spinal cord injury + pressure ulcers)	Inpatient	80%
Citty et al.	2017	Quantitative descriptive study	USA	Mixed diagnoses	Inpatient	60%
Citty et al.	2020	Quantitative descriptive study	USA	Mixed diagnoses	Inpatient	40%
Collins et al.	2019	Quantitative descriptive study	UK	Older adults	Inpatient	100%
Cornejo‐Pareja et al.	2021	Non‐randomized study	Spain	Mixed diagnoses	Outpatient	100%
Cruz‐Jentoft et al.	2008	Non‐randomized study	Spain	Older adults	Inpatient	100%
Daud et al.	2012	Randomized controlled trial	USA	Kidney disease	Inpatient	80%
de Luis et al.	2008	Randomized controlled trial	Spain	Malignancy	Outpatient	60%
de Luis et al.	2015	Non‐randomized study	Spain	Mixed diagnoses	Inpatient	80%
de Oliveria Faria et al.	2021	Quantitative descriptive study	Brazil	Malignancy	Outpatient	100%
Dedeyne et al.	2018	Quantitative descriptive study	Belgium	Older adults	Outpatient	80%
den Uijl et al.	2015	Quantitative descriptive study	Netherlands	Older adults	Mixed	100%
Dhuibhir et al.	2018	Quantitative descriptive study	Ireland	Mixed diagnoses	Inpatient	100%
Doll‐Shankaruk et al.	2008	Quantitative descriptive study	Canada	Older adults	Inpatient	100%
Enriquez‐Fern et al.	2021	Quantitative descriptive study	Canada	Malignancy	Outpatient	80%
Faccio et al.	2021	Randomized controlled trial	Brazil	Malignancy	Outpatient	60%
Fearon et al.	2003	Randomized controlled trial	UK	Malignancy	Outpatient	60%
Fiatarone et al.	2000	Randomized controlled trial	USA	Older adults	Inpatient	60%
Forli et al.	2001	Randomized controlled trial	Norway	Lung disease	Outpatient	100%
Gazotti et al.	2003	Randomized controlled trial	Belgium	Older adults	Mixed	80%
Ginzburg et al.	2018	Quantitative descriptive study	Israel	Older adults	Outpatient	80%
Gosney et al.	2003	Quantitative descriptive study	UK	Older adults	Inpatient	80%
Grass et al.	2015	Non‐randomized study	Switzerland	Malignancy	Outpatient	100%
Grönstedt et al.	2020	Randomized controlled trial	Sweden	Older adults	Inpatient	60%
Hanai et al.	2018	Randomized controlled trial	Japan	Malignancy	Mixed	60%
Hashizume et al.	2019	Quantitative descriptive study	Japan	Mixed diagnoses	Outpatient	80%
Hertlein et al.	2018	Non‐randomized study	Germany	Malignancy	Mixed	80%
Ho Norshariza et al.	2017	Quantitative descriptive study	Malaysia	Malignancy	Outpatient	0%
Hogan et al.	2019	Randomized controlled trial	Australia	Malignancy	Outpatient	100%
Hopanci Bicakli et al.	2017	Non‐randomized study	Turkey	Malignancy	Outpatient	80%
Huang et al.	2020	Randomized controlled trial	China	Malignancy	Outpatient	40%
Hübner et al.	2012	Randomized controlled trial	Switzerland	Malignancy	Outpatient	80%
Hulsbaek et al.	2021	Randomized controlled trial	Denmark	Older adults	Mixed	80%
Hung et al.	2005	Randomized controlled trial	Taiwan	Kidney disease	Outpatient	20%
Ida et al.	2017	Randomized controlled trial	Japan	Malignancy	Mixed	40%
Imamura et al.	2021	Non‐randomized study	Japan	Malignancy	Mixed	60%
Ishiki et al.	2013	Randomized controlled trial	Japan	Malignancy	Inpatient	20%
Jackson et al.	2015	Randomized controlled trial	UK	Kidney disease	Outpatient	60%
Jeloka et al.	2013	Randomized controlled trial	India	Kidney disease	Outpatient	40%
Jobse et al.	2015	Randomized controlled trial	Germany	Older adults	Inpatient	60%
Jukkola et al.	2005	Non‐randomized study	Australia	Older adults	Inpatient	100%
Karlsson et al.	2021	Randomized controlled trial	Sweden	Older adults	Inpatient	40%
Keithley et al.	2002	Randomized controlled trial	USA	Other (HIV‐infection)	Outpatient	80%
Kobayashi et al.	2017	Non‐randomized study	Japan	Malignancy	Outpatient	100%
Kong et al.	2018	Randomized controlled trial	Korea	Malignancy	Mixed	60%
Kraft et al.	2012	Randomized controlled trial	Germany	Older adults	Outpatient	0%
Lad et al.	2005	Mixed methods study	UK	Older adults	Inpatient	20%
Lambert et al.	2014	Non‐randomized study	Australia	Mixed diagnoses	Inpatient	60%
Lammel Ricardi et al.	2013	Non‐randomized study	Brazil	Mixed diagnoses	Inpatient	100%
Lauque et al.	2000	Randomized controlled trial	France	Older adults	Inpatient	20%
Laviano et al.	2020	Randomized controlled trial	Multiple countries	Malignancy	Outpatient	60%
Lawson et al.	2000	Non‐randomized study	UK	Other (orthopedic patients)	Inpatient	80%
Lawson et al.	2021	Randomized controlled trial	Canada	Malignancy	Outpatient	80%
Lidoriki et al.	2020	Quantitative descriptive study	Greece	Malignancy	Outpatient	100%
Liljeberg et al.	2019	Non‐randomized study	Sweden	Mixed diagnoses	Outpatient	100%
Lombard et al.	2014	Non‐randomized study	Netherlands	Older adults	Inpatient	80%
Malafarina et al.	2021	Non‐randomized study	Spain	Older adults	Inpatient	100%
Mantovani et al.	2004	Non‐randomized study	Italy	Malignancy	Outpatient	60%
Martin et al.	2019	Non‐randomized study	Spain	Older adults	Mixed	80%
Mayr et al.	2016	Randomized controlled trial	Germany	Other (older adults; Diabetes Mellitus)	Outpatient	100%
Mayr et al.	2000	Non‐randomized study	Germany	Mixed diagnoses	Outpatient	80%
McCormick et al.	2003	Non‐randomized study	Ireland	Older adults	Inpatient	20%
McDermott et al.	2003	Non‐randomized study	UK	Other (HIV‐infection)	Outpatient	80%
McMurdo et al.	2009	Randomized controlled trial	UK	Older adults	Outpatient	40%
Meade	2007	Non‐randomized study	Australia	Kidney disease	Outpatient	20%
Miller et al.	2005	Randomized controlled trial	Australia	Older adults	Inpatient	40%
Myers et al.	2010	Quantitative descriptive study	UK	Other (cystic fibrosis)	Outpatient	40%
Nasrah et al.	2020	Quantitative descriptive study	Canada	Malignancy	Outpatient	20%
Neoh et al.	2020	Non‐randomized study	Malaysia	Malignancy	Outpatient	100%
Olde Rikkert et al.	2015	Non‐randomized study	Multiple countries	Older adults	Mixed	100%
Palma‐Milla et al.	2016	Randomized controlled trial	Spain	Malignancy	Mixed	40%
Pastore et al.	2014	Randomized controlled trial	Brazil	Malignancy	Outpatient	40%
Patursson et al.	2021	Randomized controlled trial	Denmark	Malignancy	Outpatient	40%
Percival et al.	2013	Non‐randomized study	UK	Malignancy	Outpatient	20%
Pison et al.	2011	Randomized controlled trial	France	Lung disease	Outpatient	80%
Planas et al.	2005	Randomized controlled trial	Spain	Lung disease	Outpatient	60%
Previtali et al.	2020	Non‐randomized study	Italy	Malignancy	Mixed	80%
Qin et al.	2021	Mixed methods study	China	Malignancy	Outpatient	80%
Roberts et al.	2003	Randomized controlled trial	UK	Older adults	Inpatient	80%
Rondanelli et al.	2020	Randomized controlled trial	Italy	Older adults	Inpatient	80%
Salamon et al.	2018	Randomized controlled trial	Australia	Kidney disease	Outpatient	20%
Sandmael et al.	2017	Randomized controlled trial	Norway	Malignancy	Outpatient	20%
Schmidt et al.	2020	Non‐randomized study	Denmark	Malignancy	Outpatient	60%
Scott et al.	2009	Non‐randomized study	USA	Kidney disease	Outpatient	100%
Seemer et al.	2020	Non‐randomized study	Germany	Older adults	Inpatient	80%
Seguy et al.	2020	Non‐randomized study	France	Older adults	Outpatient	80%
Sharma et al.	2002	Randomized controlled trial	India	Kidney disease	Outpatient	40%
Shirakawa et al.	2012	Non‐randomized study	Japan	Digestive system disease	Mixed	60%
Skladany et al.	2020	Quantitative descriptive study	Slovakia	Digestive system disease	Outpatient	100%
Smith et al.	2020	Randomized controlled trial	UK	Older adults	Outpatient	60%
Solheim et al.	2017	Randomized controlled trial	Norway and UK	Malignancy	Outpatient	20%
Stange et al.	2013	Randomized controlled trial	Germany	Older adults	Inpatient	60%
Steiner et al.	2003	Randomized controlled trial	UK	Lung disease	Outpatient	80%
Storck et al.	2020	Randomized controlled trial	Switzerland	Malignancy	Outpatient	40%
Stow et al.	2015	Randomized controlled trial	UK	Older adults	Inpatient	20%
Taib et al.	2021	Quantitative descriptive study	UK	Older adults	Inpatient	100%
Tanaka et al.	2018	Non‐randomized study	Japan	Malignancy	Outpatient	100%
Tanaka et al.	2021	Randomized controlled trial	Japan	Malignancy	Outpatient	80%
Trachootham et al.	2015	Non‐randomized study	Thailand	Malignancy	Outpatient	80%
van der Berg et al.	2015	Randomized controlled trial	Netherlands	Mixed diagnoses	Inpatient	60%
van der Meij et al.	2010	Randomized controlled trial	Netherlands	Malignancy	Outpatient	80%
Verma et al.	2000	Non‐randomized study	UK	Digestive system disease	Outpatient	80%
Verma et al.	2001	Randomized controlled trial	UK	Digestive system disease	Outpatient	40%
Vermeeren et al.	2004	Randomized controlled trial	Netherlands	Lung disease	Inpatient	80%
Wall et al.	2020	Non‐randomized study	New Zealand	Digestive system disease	Outpatient	60%
Wan et al.	2021	Mixed methods study	China	Malignancy	Outpatient	100%
Weenen et al.	2014	Quantitative descriptive study	Multiple countries	Mixed diagnoses	Mixed	100%
Wengstrom et al.	2009	Non‐randomized study	Sweden	Older adults	Mixed	80%
Wong et al.	2021	Quantitative descriptive study	Singapore	Mixed diagnoses	Mixed	100%
Wu et al.	2013	Randomized controlled trial	Taiwan	Kidney disease	Outpatient	40%
Xie et al.	2021	Randomized controlled trial	China	Malignancy	Outpatient	80%
Young et al.	2018	Non‐randomized study	Australia	Older adults	Inpatient	100%
Zak et al.	2009	Randomized controlled trial	Poland	Older adults	Mixed	80%
Zhang et al.	2022	Randomized controlled trial	China	Malignancy	Outpatient	60%

^a^ Inpatient, e.g., hospital, nursing home.

## Appendix 3 Examples of Coding

The terms used for oral nutritional supplements categorized as “oral nutritional supplement” or equivalents, other than “oral nutritional supplement,” and subcategories among the 137 articles.

**Table:**

“Oral nutritional supplement”	Other than “oral nutritional supplement”
Oral nutritional supplement(s) (*n* = 32), oral nutrition supplement(s) (*n* = 4), oral nutritional supplementation (*n* = 13), commercial oral nutritional supplements (*n* = 1), complete oral nutritional supplements (*n* = 1), ONS (*n* = 1), nutrient‐ and energy‐dense oral nutritional supplement (*n* = 1)	*Terms indicating the supplement contains nutrients*: Nutritional supplement(s) (*n* = 12), nutritional supplementation (*n* = 4), nutritional supplement drinks (*n* = 1), ready‐made liquid nutritional supplements (*n* = 1), dietary supplements (*n* = 1), nutrition supplement (*n* = 1), nutritional drink supplement (*n* = 1), nutritional support (*n* = 1), oral nutritional support (*n* = 2), nutritional formula (*n* = 1), oral nutritional formula (*n* = 1)
	*Terms indicating it is a supplement of some kind*: Oral supplement(s) (*n* = 5), oral supplementation (*n* = 2), supplements (*n* = 1), active supplements (*n* = 1), enrichment module (*n* = 1)
	*Terms indicating the supplement is enriched with protein, carbohydrates, and/or energy*: Protein supplementation (*n* = 1), protein‐rich nutritional supplements (*n* = 1), protein‐rich oral supplementation (*n* = 1), oral protein supplements (*n* = 1), liquid protein supplement (*n* = 1), leucine‐rich supplement (*n* = 1), high‐protein supplementation (*n* = 1), collagen peptide supplementation (*n* = 1), arginine‐enriched oral nutrition supplement (*n* = 1), protein‐energy supplement (*n* = 1); protein‐energy oral supplementation (*n* = 1); high‐protein, high‐calorie oral nutritional supplementation (*n* = 1); high‐calorie supplementation (*n* = 1), carbohydrate‐rich supplement (*n* = 1)
	*Terms indicating the supplement is disease specific*: Diabetes‐specific oral nutritional supplement (*n* = 1), intradialytic oral nutritional supplementation (*n* = 1), oral diabetes‐specific supplement (*n* = 1), oral elemental nutritional supplement (*n* = 1), peridialytic oral supplements (*n* = 1), elemental diet (*n* = 1)
	*Terms indicating the supplement affects the immune system*: Immunonutrition (*n* = 1), immunomodulating nutritional formula (*n* = 1), immune‐enhancing oral formulas (*n* = 1), immunonutrition supplements (*n* = 1), pharmaconutritional support (*n* = 1), preoperative immunonutrition (*n* = 1), oral immunonutrition (*n* = 2)
	*Terms indicating the supplement is home‐made*: Home‐made oral supplement (*n* = 1), homemade milk‐based oral supplement (*n* = 1), non‐industrialized nutritional supplement (*n* = 1)
	*Terms indicating the supplement is made for medical purposes*: Medical nutrition (*n* = 1), medical food (*n* = 1), targeted medical nutrition (*n* = 2), muscle‐targeted food for special medical purposes (*n* = 1)
	*Other terms*: Conventional commercial supplements and MedPass (*n* = 1), sip‐feed supplement (*n* = 2), food supplement (*n* = 1), non‐protein calorie supplement (*n* = 1), ice‐cream based supplement (*n* = 1), nutri‐jelly (*n* = 1)

### Variable Coding of Terms of Adherence

If several terms for adherence were used, the following order was applied for variable coding: 1. Adherence term used in title, 2. Adherence term used in the abstract, if there were more than one term mentioned in the title or abstract, all of these were reported. 3. The first term mentioned in the method section. When no term for adherence was mentioned in the title, abstract, or the method, the term was searched for in the remaining text.

### Variable Coding of Type of Oral Nutritional Supplement

When articles did not clearly report the type of ONS used (e.g., milk‐based or juicy), but instead the ONS brand, the type of ONS was determined by searching the brand of the ONS on the internet.
